# Pharmacological Effects and Immune Mechanisms of Oriental Medicines in Restoring Male Infertility

**DOI:** 10.3390/ijms26104642

**Published:** 2025-05-13

**Authors:** Ning Qu

**Affiliations:** 1 Department of Immunoregulation, Institute of Medical Science, Tokyo Medical University, Tokyo 160-8402, Japan; quning@tokyo-med.ac.jp or quning@tokai.ac.jp; Tel.: +81-3-3351-6141; Fax: +81-3-3351-6250; 2 Department of Anatomy, Tokai University School of Medicine, Isehara 259-1143, Japan

**Keywords:** infertility, oriental medicine, molecular immune suppress, spermatogenesis

## Abstract

Male infertility can be the result of many factors, including pathologies in the motility and formation of sperm and obstructions in the genitourinary system. Oriental medicine, using multiple components to address various targets and enhance therapeutic effectiveness, has been used to treat male infertility for thousands of years. Given the complex pathological mechanisms of aspermatogenesis, this multi-target approach in oriental medicine is believed to positively impact the prevention of male infertility. Efforts have been made to evaluate the pharmacological properties of many herbs. However, the testicular immune responses and mechanisms of these oriental medicines remain unclear from a modern scientific perspective. Some studies reviewed here have reported on the recovery of spermatogenesis in infertility, the available research that evaluates the efficacy of oriental medicines in the treatment of aspermatogenesis and briefly summarizes the available rodent and human data on facilitating immunological differences in male infertility. These findings augment the current understanding of the immune mechanisms of oriental medicine as a treatment solution for male infertility.

## 1. Introduction

Infertility is a global health issue affecting up to 15% of couples, with male fertility contributing to at least 50% of cases [1,2,3]. The established causes of male infertility range from pre-testicular to testicular to post-testicular. The pretesticular causes include hypogonadotropic hypogonadism, chronic alcoholism, cigarette smoking, drugs, and substance abuse. The testicular causes include congenital anomalies, cryptorchidism, orchitis, testicular tumors, testicular torsion/detorsion, varicocele, radiotherapy, and chemotherapy. The post-testicular causes include obstruction or absence of the vas deferens or ejaculatory duct, hypospadias, and erectile dysfunction [4,5,6,7]. Modern medicine emphasizes identifying the primary causes and utilizing various hormones in treatment [8]. The main approaches include general treatment, such as lifestyle change, endocrine therapy (gonadotropin-releasing hormone (GnRH) agonists, gonadotropins, anti-estrogens, and aromatase inhibitors), anti-infective therapy, antioxidant therapy, antibiotics, corticosteroids, vitamins, minerals (such as zinc) and amino acids (such as arginine), surgical intervention, and assisted reproductive technology (ART) [9,10]. Gene therapy is still in the research phase and has not yet been implemented in clinical practice [11]. Although ART has significantly improved conception rates among infertility patients, it has not fundamentally enhanced sperm quality.

Infertility is not an isolated condition but arises from multiple diseases and factors [12,13]. Currently, oriental medicine has been used to treat male infertility for thousands of years. A key characteristic of oriental medicine is the use of multiple components to address various targets and enhance therapeutic effectiveness. Given the complex pathological mechanisms of aspermatogenesis, this multi-target approach in oriental medicine is believed to positively impact the prevention of male infertility [14]. The treatment of spermatogenesis with various oriental medicines differs from modern medicine. The fundamental concept in oriental medicine is that two opposite yet complementary forces underline all natural phenomena, Yin and Yang, which are used to characterize both events and physical processes. For example, Yin represents cold, consolidation, and the quality of being at rest, while Yang represents heat, expansion, and activity. In oriental medicine, the state of homeostasis may be regarded as a perfect balance of Yin and Yang, and disease is viewed as the result of disharmony or imbalance. Although oriental medicine treatments generally operate at a more complex theoretical level, the balance of Yin and Yang in the body is the ultimate therapeutic goal [15]. Furthermore, the active principle underlying physiology and metabolism in the human body is *qi*, which is the vital energy that permeates the universe. Each organ has its own type of *qi* that enables it to perform its functions. In some cases, these functions coincide with our modern Western understanding of organ function. The organ most involved in the oriental medicine understanding of male infertility and erectile dysfunction is the kidney. The kidney stores the essence *qi* (stagnation of circulation of vital energy), which roughly corresponds to our modern concept of male and female gametes. Robust kidney *qi* enhances sperm motility, whereas a decline in sperm motility is associated with kidney hypofunction [15]. Insufficient kidney Yang impairs kidney Yin’s ability to generate essence and *qi*, leading to reduced sperm count, decreased sperm motility, and infertility. Several studies have identified the active components of herbal medicines that nourish the kidney, support blood circulation in the reproductive system, and regulate testosterone secretion [16]. They have been shown to significantly improve sperm motility in animal studies and have also been shown to have positive effects in enhancing sperm parameters in infertile men [17,18].

In recent years, efforts have been made to evaluate the pharmacological properties of many herbs. However, the testicular immune responses and mechanisms of these oriental medicines remain unclear from a modern scientific perspective. This review aims to summarize some infertility types with insufficient treatment effects in Western medicine and to examine studies evaluating the use of traditional herbal formulas rather than individual herbal medicines in treating this condition. Our findings enhance the current understanding of the potential of oriental medicine as a treatment option for male infertility.

## 2. Male Infertility with Insufficient Treatment Effects in Western Medicine

**Figure 1:**
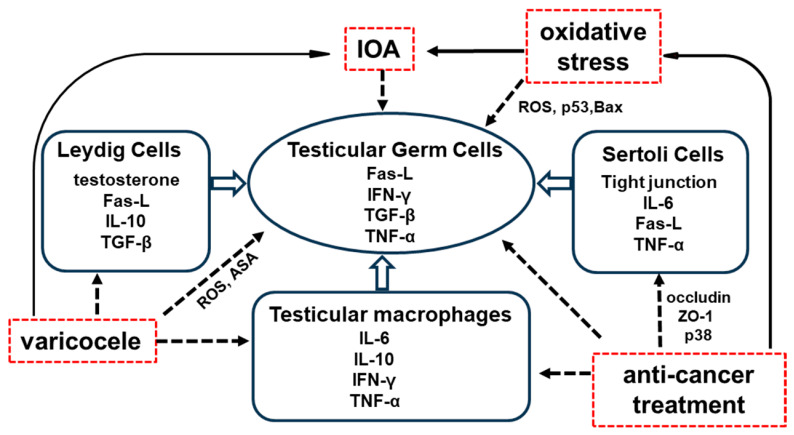
Immune molecular mechanisms in male infertility cases. Several male infertility cases deal with insufficient treatment or effects of using Western medicine approaches. Abbreviations: anti-sperm antibodies: ASA; B-cell lymphoma 2: Bcl-2; Bcl-2 associated X protein: Bax; Fas ligand: Fas-L; interferon-γ: IFN-γ; interleukin: IL; idiopathic oligoasthenozoospermia: IOA; reactive oxygen species: ROS; transforming growth factor β: TGF-β; tumor necrosis factor α: TNF-α.

The most recognized immunological privilege mechanism in the testis is the blood–testis barrier (BTB), which is formed by the junctions of adjacent Sertoli cells. This barrier restricts access to germ cell antigens for interstitial immune cells and protects against antibody attacks from the interstitium [19]. Immunological privileges outside the BTB involve the secretion of immunosuppressive factors primarily by macrophages, Sertoli cells, peritubular cells, and Leydig cells [20,21] (see Figure 1 and Table 1). Although the mechanisms of immune privilege in male reproductive organs are well studied, a deeper understanding of the underlying immune responses and mechanisms of male infertility is essential for advancing treatment.

Around 30–40% of infertile men fall into the category of idiopathic oligoasthenozoospermia (IOA), characterized by a low sperm count (16 × 10^6^ spermatozoa per milliliter of semen) and poor sperm quality (<30% of spermatozoa exhibiting progressive motility) [38,50]. Common causes of IOA include endocrine factors, reproductive tract infections, chromosomal abnormalities, cryptorchidism, varicocele, systemic diseases, and various genetic, metabolic, and immune dysfunctions. IOA is not an independent condition. Rather, it results from a combination of multiple diseases and factors [12,13]. Although various factors contribute to the pathogenesis of IOA, the underlying mechanisms remain unclear (see Figure 1). Consequently, Western medicine faces certain limitations. Its clinical efficacy is often suboptimal, and finding a cure for IOA has long been a significant challenge in the medical field. Modern medical treatments for IOA include general treatment, endocrine therapy (such as GnRH agonists, gonadotropins, antiestrogens, and aromatase inhibitors), antioxidant therapy, antibiotics, corticosteroids, vitamins (e.g., vitamin E), minerals (such as zinc), amino acids (such as arginine), anti-infective therapy, surgical interventions, and ART [9,10]. However, these strategies are often associated with poor outcomes. From the databases consulted, there is an urgent need to investigate the molecular mechanisms underlying IOA and identify effective treatments for IOA-related infertility.

There is growing evidence that oxidative stress in seminal fluid is a key factor in poor semen quality, including in IOA [51]. Oxidative stress, characterized by an imbalance between reactive oxygen species (ROS) and antioxidant levels, is the primary cause of infertility in men. The testis contains high levels of polyunsaturated fatty acids, making them an ideal substrate for ROS generation. Elevated ROS levels can lead to infertility through mechanisms such as lipid peroxidation, DNA damage, enzyme inactivation, and protein oxidation in spermatozoa (see Figure 1). These processes compromise sperm functionality and reduce sperm motility and viability [23]. Studies indicate that 30–80% of infertile men exhibit elevated ROS levels, which can damage DNA, as well as the proteins and lipids of sperm. This damage can interfere with the capacitation processes necessary for successful fertilization [52,53,54,55]. Furthermore, in our daily lives, genital heat stress, such as sitting in a hot bath or car for an extended period, is associated with significantly higher scrotal temperatures. This condition is a major factor contributing to the impairment of male reproductive health, resulting in the downregulation of spermatogenesis and declines in semen volume, sperm motility, and sperm morphology [51]. It has been demonstrated that body weight, testicular weight, sperm count, and sperm motility were significantly reduced in male mice subjected to heat stress in a 43 °C water bath for 10 min twice per day over a period of five weeks [56,57]. Germ cell death and decreased sperm motility resulting from heat stress appear to be caused by oxidative stress and apoptosis. These processes involve ROS, the tumor suppressor protein p53, nitric oxide synthase (NOS), and translocation of the pro-apoptotic factor Bax [31,32] (see Figure 1 and Table 1). The body’s antioxidant systems, comprising enzymatic factors such as superoxide dismutase, catalase, and glutathione peroxidase, along with non-enzymatic factors such as low-molecular-weight compounds (e.g., glutathione, N-acetylcysteine, vitamins E, A, and C, coenzyme Q10, carnitines, myo-inositol, and lycopene) and essential nutrients (selenium, zinc, and copper), play a critical role in protecting against oxidative stress [55]. Of 78 (20- to 40-year-old) patients’ examinations, each subject had a semen leucocyte concentration of less than 1 mln cells/mL and additionally met one or more of the following inclusion criteria: total sperm count in the ejaculate <39 mln/mL, progressive sperm motility <32%, and/or a morphologically pathological sperm level >96%. All patients took a combination supplement twice daily for 6 months; each dose contained 1725 mg L-carnitine fumarate, 500 mg acetyl-L-carnitine, 90 mg vitamin C, 20 mg coenzyme Q10, 10 mg zinc, 200 µg folic acid, 50 µg selenium, and 1.5 µg vitamin B12 (Proxeed^®^ Plus, Sigma-Tau, Rome, Italy) [58]. However, their results show that the total sperm count in ejaculate only increased 1.9 mln/mL, sperm progressive motility only increased 9%, and total sperm motility only increased 7.4% [58]. Many urologists prescribe oral antioxidants to address infertility because they enhance seminal plasma clearance and lower ROS levels in the semen, but currently, there are no effective medications for IOA [59].

Varicocele, another common cause of IOA, characterized by the enlargement of the pampiniform venous plexus within the spermatic cord, is generally acknowledged as the most common but curable cause of male infertility. Varicocele can affect spermatogenesis in many ways, e.g., through increased testicular temperature, increased intratesticular pressure, hypoxia due to attenuation of blood flow, the reflux of toxic metabolites from the adrenal glands, and hormonal profile abnormalities [60]. Varicocele has also been associated with increased oxidative stress, especially in the gonads [48]. The incidence of varicocele is approximately 15%, affecting more than 40% of men in infertile couples [61,62,63]. The precise mechanism by which varicocele leads to infertility remains unclear. However, factors such as the testicular immune response, including the production of anti-sperm antibodies (ASA), activation of inflammatory pathways (increase in the pro-inflammatory cytokines such as interleukin (IL)-1 and tumor necrosis factor (TNF)-alpha in the seminal plasma, testicular tissue, and even peripheral blood), and disruption of the BTB (abnormal permeability of proteins, such as claudin-11), may contribute to the development of varicocele-related infertility [22] (see Figure 1 and Table 1). Currently, there are no effective medications for most varicoceles, and the primary treatment approach is surgery. However, some patients may still struggle to regain their natural fertility after surgery. Medications are often considered the first-line option for patients with low spermatogenesis and varicocele who do not undergo surgical treatment, although their endocrine effects are debated. Selective estrogen receptor modulators (SERMs), such as clomiphene and tamoxifen, are commonly used as empirical treatments for male infertility. These medications function by blocking estrogen receptor (ER) activity, which promotes luteinizing hormone (LH) production and stimulates sperm production. Although the use of SERMs may have positive effects on pregnancy outcomes, their overall efficacy remains uncertain [37]. In clinical practice, antioxidants, such as kallikrein, L-carnitine, anthocyanin, silymarin, chrysin, selenium, and resveratrol, are also used in the treatment of varicocele. However, current studies do not provide sufficient evidence to support the use of these substances for treating varicocele [64]. Therefore, the development of safe medical treatments for varicocele with fewer side effects is still in demand.

On the other hand, the risk of anti-cancer treatment-induced oligoasthenozoospermia and azoospermia increases with the development of chemotherapy and radiotherapy, which significantly improved survival rates in children with cancer. More than 70% of patients survive childhood cancer. However, these treatments may cause irreversible impairment of reproductive function. Infertility affects 30% of long-term survivors of childhood acute myelogenous leukemia (AML), leaving them unable to father children, even with the use of assisted reproductive techniques [65,66]. The testis is more sensitive to anti-cancer drugs and radiation than the ovary, and both anti-cancer agents and radiation therapy are often used in combination with hematopoietic cell transplantation pretreatment methods, further increasing the risk of testicular injury. Cytotoxic agents, including alkylating agents such as cyclophosphamide (CP), ifosfamide, and chlorambucil, as well as procarbazine, cisplatin, and vinblastine, can cause long-lasting or permanent damage to germ cells, resulting in oligozoospermia or azoospermia (see Figure 1). In fact, azoospermia is permanent in 90% of men treated with CP at doses greater than 7.5 g/m^2^ [67]. Germ cells are also susceptible to fractionation by radiotherapy, with doses exceeding 1.2 Gy, leading to permanent azoospermia [68]. Following treatment with total body irradiation (9.9 or 13.2 Gy) and CP for bone marrow transplantation, azoospermia was observed in 85% of adult male patients [69]. Testicular toxicity is one of the main side effects of CP, with oxidative stress damage being the primary cause of testicular spermatogenesis disorders [70] (see Figure 1 and Table 1). Studies have confirmed that oxidative stress can lead to an abnormal expression of tight junctions, the key components of the BTB, and related proteins by activating the p38 MAPK pathway [71,72]. It is well-known that occludin and ZO-1 play crucial roles in maintaining the normal function of tight junctions. Fibrous actin (F-actin), a polymer of actin filaments, is associated with ZO-1 and is essential for the integrity of tight-junction network structures. Li et al. [36] demonstrated that CP could reduce the expression of occludin, ZO-1, and F-actin, while increasing the expression of p38, significantly inducing BTB damage in rats (see Figure 1 and Table 1). Although advancements in molecular biology, such as the assessment of sperm mRNAs, microRNAs, histone modifications, and DNA methylation, have been made, there is a lack of innovative approaches that could enhance the evaluation of sperm health following anti-cancer therapy. Currently, the only established option is the cryopreservation of sperm prior to treatment. Sperm cryobanking allows patients to preserve sperm before undergoing gonadotoxic therapies, facilitating future assisted-reproduction techniques, such as in vitro fertilization or intracytoplasmic sperm injection [65]. Although post-pubertal males can cryopreserve their own sperm, there are currently no options available to prevent infertility in pre-pubertal boys undergoing chemotherapy and/or radiotherapy. Hormonal protection of germ cells using GnRH agonists has shown success in rodent models [73] but has not been effective in humans [74]. Over the past decade, fertility preservation has emerged as an area of increasing awareness among researchers, clinicians, and patients [40]. However, there is limited information regarding therapies for anticancer-induced male infertility. Although some attempts at medical treatment for male factor infertility have been reported, adequately controlled studies of potential therapeutic agents often fail to demonstrate significant improvements in fertility.

## 3. Pharmacological Effects and Immune Mechanisms of Oriental Medicines

Oriental medicine posits that sperm production and maturation are closely linked to kidney deficiency, viewing the kidney as a congenital foundation that stores essence and governs growth, development, and reproduction. Robust kidney *qi* (stagnation of circulation of vital energy) enhances sperm motility, whereas a decline in sperm motility is associated with kidney hypofunction. Insufficient kidney Yang impairs the kidney Yin’s ability to generate essence and *qi*, leading to reduced sperm count, decreased sperm motility, and infertility.

### 3.1. Varicocele

In an animal model examination reports, Jiawei Runjing Decoction (JWRJD) by adding two traditional Chinese medicines [*Homo Sapiens* and *Eupolyphaga seu Steleophaga*] (see Table 2) into Runjing Decoction, could be more powerful for promtoing blood circulation and clear collaterals, nourish kidney Yang and other functions to improve the proliferation of mouse germ cells, and increase the number of sperms [49,75]. In another experimental varicocele model study, Peng et al. [39] orally administered Zishen Yutai Pill (ZYP), which contains 15 traditional Chinese medicines (see Table 2). Their results demonstrated that ZYP significantly improved sperm motility, reduced the sperm DNA fragmentation index, and alleviated testicular tissue damage caused by experimental varicocele in rats. Furthermore, the mRNA and protein expression levels of nucleotide-binding oligomerization domain-like receptor family pyrin (NLRP) inflammasome components, specifically NLRP3, ASC, and caspase-1, were significantly downregulated in rats treated with ZYP. The serum level of IL-1β was also significantly reduced in ZYP-treated rats, suggesting that ZYP may act as an NLRP3 inhibitor, suppressing the IL-1β-dependent inflammatory response in the testicles of varicocele-affected rats (see Table 1 and Figure 1).

**Figure 2:**
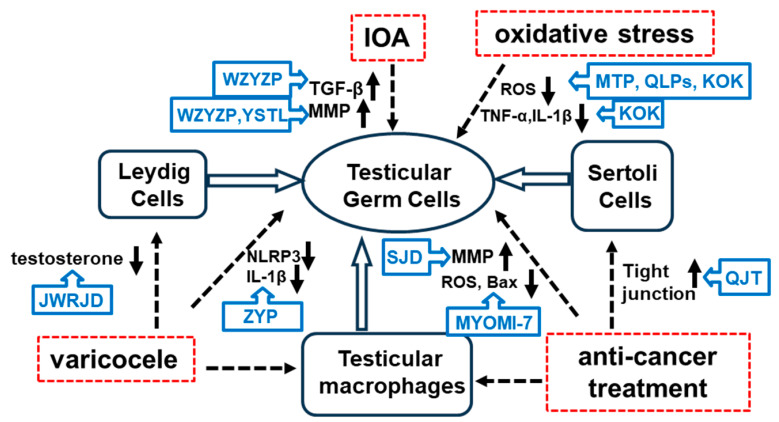
Pharmacological effects and immune mechanisms of oriental medicines. ↑ indicates an increase and ↓ indicates a decrease.

Furthermore, in human patient reports, Liu et al. [49] applied JWRJD to 58 cryptozoospermic patients with varicocele who were observed for 3 months and found that JWRJD may promote spermatogenesis in cryptozoospermic patients with varicocele closely related to the sex hormone mechanism and improve the testicular microenvironment, especially for patients > 35-year-old and having grade III varicocele (see Table 2 and Figure 2). Importantly, they demonstrated that the testicle volume, FSH, prolactin, neutral α-glucosidase, citric acid, and zinc in the JWRJD treatment group were higher than those in the tamoxifen treatment group (all *p* < 0.05), although tamoxifen treatment also significantly increased the total sperm count.

Another Chinese research group [30] investigated the mechanism of action of Taohong Siwu Decoction (THSWD) (see Table 2), which is believed to remove blood stasis, clear collaterals, and nourish the blood, effectively alleviating symptoms such as testicular swelling, perineal swelling and pain, and the bulging of testicular tendons in varicocele-associated male infertility. Using network pharmacology and molecular docking, they demonstrated that THSWD regulates varicocele-associated male infertility through multiple compounds and targets. Their findings indicate that the mechanism is closely linked to the inflammatory response, ROS damage, and blood vessel function.

In oriental medicine, varicocele cryptozoospermia is considered to be linked to the deficiency syndrome of “*qi*-stagnation”, “*blood*-stasis” (coagulation of blood), and kidney Yang [76]. The above research terms utilized showed that tonification of the kidney is the fundamental theory underlying the treatment of varicocele-induced male infertility in oriental medicine. The studies about JWRJD and THSWD showed that correcting “*qi*-stagnation”, “*blood*-stasis”, and the tonification of kidney Yang are important points in the treatment of varicocele. Treatment with JWRJD demonstrated that it can increase testicular volume, decrease follicle-stimulating hormone (FSH), decrease testicular vein width, and increase seminal plasma zinc levels, although investigation of the testicular immune response in the pathogenesis of varicocele-mediated infertility was not conducted. On the other hand, the study on THSWD identified that it may exert therapeutic effects through the HIF-1, PI3K-Akt, and Relaxin signaling pathways. This study provides scientific evidence for subsequent experimental research and rational clinical administration of THSWD for the treatment of varicocele-induced male infertility. Furthermore, the experimental varicocele model study of ZYP is so similar to and supports the previous study that showed the involvement in the activation of potential inflammatory pathways, such as the NLRP3 pathway in varicocele-induced infertility [63]. This animal study is noteworthy for its exploration of the immune and inflammatory factors associated with varicocele-related cryptozoospermia (see Table 1 and Figure 2).

### 3.2. Oxidant Stress

Herbal therapy is increasingly being considered as a preventive measure against infertility, as these natural antioxidants can help mitigate the harmful effects of oxidative stress [41]. There are some animal model examination reports about the pharmacological effects and immune mechanisms of oriental medicines in oxidative stress-induced infertility.

MOTILIPERM (MTP), a formulation made from a mixture of three medicinal plants (see Table 2), was developed to treat male infertility in South Korea [28]. Karna et al. investigated the mechanisms underlying the effects of MTP on testicular dysfunction induced by immobilization stress in rats. They demonstrated that MTP could reduce oxidative stress by significantly increasing testicular superoxide dismutase (SOD) levels and decreasing malondialdehyde (MDA) and ROS/RNS levels. Additionally, they measured the markers of testicular apoptosis and observed a downregulation of cleaved caspase-3 and B-cell lymphoma 2 (Bcl-2)-associated X protein (Bax) levels, alongside increased levels of pro-caspase-3 and Bcl-2, as well as upregulated germ cell proliferation in the testes (see Table 1 and Figure 2). This research group also demonstrated that MTP could restore testicular function by decreasing ROS-induced endoplasmic reticulum stress and germ cell apoptosis while upregulating testosterone synthesis in a varicocele-induced rat model [33] (see Table 1 and Figure 2).

Qilin Pills (QLPs), a classic traditional Chinese medicine formula, contain 15 types of herbal medicines (see Table 2). Clinical trials have shown that QLPs effectively improve semen quality. Zhang et al. [42] demonstrated that QLPs have a therapeutic effect in a rat model of oligoasthenozoospermia by restoring levels of FSH and LH, along with reduced levels of oxidative stress products (see Table 1 and Figure 2).

Kyung-Ok-Ko (KOK) (see Table 2), also known as Qiong-yu-gao in China, is a well-known traditional medicinal formula in both Korean and Chinese medicine and has long been used to invigorate essential *qi* [77]. Some studies have demonstrated that KOK treatment significantly restores the morphological appearance of seminiferous tubules and the epithelium. Additionally, significant increases in sperm count and motility were observed in mice following heat exposure. [78]. KOK was also found to inhibit the expression of IL-1β, a proinflammatory cytokine, thus showing anti-inflammatory properties [79], inhibiting TNF-α secretion by inhibiting IL-1 secretion, and having anti-inflammatory activity [35] (see Table 1 and Figure 2). The above research suggests that KOK improves heat-induced male infertility through its antioxidant, anti-inflammatory, anti-apoptotic, and protective effects on spermatogenesis.

These experimental studies have researched and proven the pharmacological effects and immune mechanisms of the three oriental medicines in oxidative stress-induced infertility. Although the compounds of these oriental medicines are different, a similar effect of correcting the imbalance between reactive oxygen species (ROS) and antioxidant levels is demonstrated in rat or mouse experimental models. Clinical studies indicate that 30–80% of infertile men exhibit elevated ROS levels [52]. However, there is no effective treatment in Western medicine currently. Based on the experience of clinicians, oriental medicine treatment is expected to create an academic foundation for oxidative stress-induced male infertility. Especially the medicine MTP, a mixture of only three medicinal plants, should have few side effects and is, therefore, likely to be applicable to clinical treatment.

### 3.3. IOA

In oriental medicine, IOA should be treated by regulating both the kidney Yin and Yang. The Wuzi Yanzong pill (WZYZP) (see Table 2) is one of the most commonly prescribed Chinese herbal formulas for treating male infertility. Originating from the renowned traditional Chinese medicine prescriptions of the Tang Dynasty, these ingredients have been widely used for an extended period to improve semen quality and address infertility. Some clinical studies have indicated that WZYZP exerts therapeutic effects in patients with IOA by significantly increasing the sperm concentration (WZYZP participants numbered 509 vs. control participants numbered 409), improving sperm motility (WZYZP participants numbered 509 vs. control participants numbered 409), and decreasing the sperm DNA fragmentation index (WZYZP participants numbered 66 vs. control participants numbered 56) compared to placebo or vitamin control [43,80,81]. Additionally, in vitro studies have demonstrated that WZYZP treatment can enhance germ cell proliferation, inhibit apoptosis, restore serum hormone levels, reduce oxidative stress-induced damage, promote spermatogenesis, and improve sperm cell quality in IOA model rats [24]. More importantly, these studies demonstrated that the WZYZP also suppressed TGF-β expression and activated the PI3K/AKT/mTOR signaling pathway, thereby promoting germ cell proliferation and inhibiting germ cell apoptosis (see Table 1 and Figure 2) [24]. It is well established that mitochondria play a crucial role in sperm motility and fertilization, primarily through glycolysis and oxidative phosphorylation, which provide the energy necessary for sperm motility [44]. Mitochondrial membrane potential (MMP), directly linked to sperm motility, serves as an indicator of mitochondrial energy status [82]. Studies have also examined correlations between impaired mitochondrial function, reduced sperm motility, and decreased reproductive ability. Lower MMP levels can lead to decreased sperm motility owing to reduced ATP production [34,45]. Shen et al. found that WZYZP restored spermatogenic functions in IOA rats, including increased sperm density, motility, viability, MMP levels, and testicular histopathology (see Table 1 and Figure 2) [81].

Several clinical trials have confirmed the efficacy of Yishentongluo decoction (YSTL) (see Table 2) as a complementary therapy for IOA. YSTL has been shown to be an effective prescription for treating male infertility associated with kidney deficiency and *blood* stasis. Other active ingredients in herbal medicines contained in YSTL also provide a well-balanced supply of minerals, antioxidants, and nutrients [83,84]. In a randomized controlled study, a total of 160 IOA patients were assigned to the YSTL group or the Levocarnitine oral solution group in a 1:1 ratio, and the treatment period was 12 weeks. They provided initial evidence for the potential mechanisms by which YSTL improves sperm motility were explored based on the assessment of MMP in spermatozoa (see Table 1 and Figure 2) [46].

It is well-known that IOA is not an independent condition and results from a combination of multiple diseases and factors. The underlying mechanisms remain unclear. Consequently, Western medicines are often associated with poor outcomes. The pharmacological effects and immune mechanisms of WZYZP and YSTL are the same as promoting spermatogenesis with increased mitochondrial function by restoring MMP levels in spermatozoa in the IOA animal model. In the above two oriental medicines, *Cuscuta chinensis (CC)*, a common compound included in WZYZP and YSTL, is well-known for its antioxidant, anti-inflammatory, anti-radiation, and immune boosting properties. Some studies have reported that the components of *CC*, *Epimedium brevicornu (EB)* and *Rehmanniae radix (RR)*, can reduce ROS, improve sperm motility, and significantly increase pregnancy rates [85]. The above oriental medicine research further proved the evidence that oxidative stress is an important factor in IOA-induced infertility, and oriental medicine is an effective treatment.

### 3.4. Anti-Cancer Treatment

Li et al. investigated whether Qiangjing tablets (QJT) (see Table 2) could contribute to the recovery from BTB dysfunction in CP-treated rats. Their results showed that the expression levels of the aforementioned indicators returned to normal after treatment with QJT, highlighting the role of QJT in regulating the expression of key proteins in the BTB and p38 MAPK pathways, thereby mitigating CP-induced BTB dysfunction and aspermatogenesis (see Table 1 and Figure 2) [36].

Sheng Jing Decoction (SJD), a traditional Chinese medicine (see Table 2), is primarily used to treat male infertility [86]. Yan et al. investigated the role of SJD in treating male infertility in mice with CP-induced oligozoospermia [47]. Their data showed that SJD treatment increased the expression of the Sertoli cell marker GATA4 and the germ cell marker TRA98 in CP-induced asthenozoospermic mice. In addition, sperm concentration and vitality were significantly enhanced following SJD treatment. Furthermore, this study demonstrated that SJD plays a crucial role in sustaining mitochondrial function and sperm motility by restoring MMP levels and preserving sperm plasma membrane integrity, both of which are impaired by CP induction (see Table 1 and Figure 2) [47].

**Table 2:** The list of oriental medicines affects male immune–reproductive system.

	Oriental Medicine	Compounds	Models	Treatment (Dosages/Duration)	Effect Sizes
Anti-cancer treatment	Goshajinkigan (TJ107) [26,27,29] (Japanese)	*Rehmanniae radix (RR)*, *Achyranthis radix*, *Corni fructus*, *Dioscoreae rhizome*, *Plantaginis semen (PS)*, *Alismatis rhizome*, *Hoelen*, *Moutan cortex*, *Cinnamomi cortex*, processed *Aconite tuber*	animal	free access to TJ107 diet containing 5.4% (*w*/*w*) extract	n = 60
				60 days	
	MYOMI-7 [25] (Korean)	*Cuscuta chinensis (CC)*, *Lycium chinense (LC)*, *Epimedium koreanum*, *Rubus coreanus*, *Morinda officinalis*, *Cynomorium songaricum*, *Cistanche deserticola*	animal	790 mg/kg/day by gavage	n = 8
				21 days	
	Qiangjing tablets (QJT) [36] (Chinese)	*Ginseng radix et rhizoma*, *Angelica sinensis radix*, *RR*, *Corni fructus*, *Lycii fructus (LF)*, *Schisandrae chinensis fructus*, *Cuscutae semen*, *PS*, *Epimedii folium*, *Common curculigo orchioides*, *Herba leonuri*	animal	0.45 g/kg/day by gavage	n = 10
				4 weeks	
	Sheng Jing Decoction (SJD) [47,86] (Chinese)	*RR*, *Astragalus membranaceus*, *Pseudostellaria heterophylla*, *Dipsacus acaulis*, *Lycium arenicolum*, *Astragalus complanatus*, *Gleditsia sinensis*	animal	33 g, 16.5, and 8.25 g/kg/day by gavage	n = 6 per group
				35 days	
Varicocele	Jiawei Runjing Decoction (JWRJD) [49,75] (Chinese)	*CC*, *Dioscorea polystachya*, *Polygonatum sibiricum*, *Epimedium brevicornu (EB)*, *Lycium barbarum*, *Eleutherococcus senticosus*, *Rhodiola crenulata*, *Cyathula officinalis*, *Citrus × aurantium*, *Hirudo*, *Homo sapiens*, *Eupolyphaga seu Steleophaga*.	animal and human	4.725 and 18.9 g/kg/day by gavage	n = 8 per group
				4 weeks	
				one dose/day	n = 58
				90 days	
	Taohong Siwu Decoction (THSWD) [30] (Chinese)	*Persicae semen*, *Carthami flos*, *RR*, *Paeoniae radix alba*, *Chuanxiong rhizoma*, *Angelicae sinensis radix*	GeneCards database, STRING database, DAVID database, RCSB database		
	Zishen Yutai Pill (ZYP) [39] (Chinese)	*CC*, *Ginseng radix et rhizoma*, *Dipsaci radix*, *Taxilli herba*, *Eucommiae cortex*, *Morindae officinalis radix*, *Cervi cornu degelatinatum*, *Codonopsis radix*, *Atractylodis macrocephalae rhizoma*, *Asini corii colla*, *LF*, *RR*, *Polygoni multiflori radix*, *Artemisiae argyi*, *Amomi fructus*	animal	1575 and 3150 mg/kg/d by gavage	n = 6 per group
				6 weeks	
Oxidant stress	Kyung-Ok-Ko (KOK) [35,77,78,79] (Korean)	*RR*, *purpurea*, *Panax ginseng*, *Poria cocos*, *LC*, *Aquilaria agallocha*, honey	animal	0.25, 0.50, and 2.00 g/kg/day by gavage	n = 8 per group
				5 weeks	
	MOTILIPERM (MTP) [28,33] (Korean)	*Rubiaceae root*, *Convol vulaceae seed*, *Liliaceae outer scales*	animal	100 and 200 mg/kg/day by gavage	n = 10 per group
				30 days	
	Qilin pills (QLPs) [42] (Chinese)	*Polygonum multijiorum*, *Herba Ecliptae*, *Eclipta prostrata*, *EB*, *CC*, *Cynomorium songaricum*, *Codonopsis pilosula*, *Curcuma aromatica*, *LC*, *Rubus idaeus*, *Dioscorea oppositifolia*, *Salvia miltiorrhiza*, *Astragalus membranaceus*, *Paeonia lactiflora*, *Citrus reticulata*, *Morus alba*	animal	1.62 and 3.24 g/kg/day by gavage	n = 10 per group
				60 days	
IOA	Wuzi Yanzong pill (WZYZP) [18,43,80,81] (Chinese)	*CC*, *LF*, *Rubi fructus*, *Schizandrae fructus*, *PS*	in vitro and animal	12.0 g/kg 2 times a day	
				7 d	
				0.635, 1.269, and 2.538 g/kg/day, by gavage	n = 12 per group
				30 days	
	Yishentongluo decoction (YSTL) [34,83,84,85] (Chinese)	*CC*, *EB*, *RR*, *Astragalus propinquus*, *Salvia miltiorrhiza*, *Cyathula officinalis*	human	1 grid granule 2 times a day	n = 80
				12 weeks	

A Korean research group investigated the therapeutic effects of MYOMI formulations on CP-induced male infertility in a mouse model. They demonstrated that treatment with MYOMI formulations reduced the CP-induced apoptosis of germ cells, as indicated by the expression levels of Bax, Bcl-2, and caspase-3, as well as oxidative stress markers, including ROS and MDA (see Table 1). Among the formulations, MYOMI-7, a Korean herbal medicine (see Table 2), showed superior results in recovering CP-induced damage to the testes and improving fertility [25].

**Figure 3:**
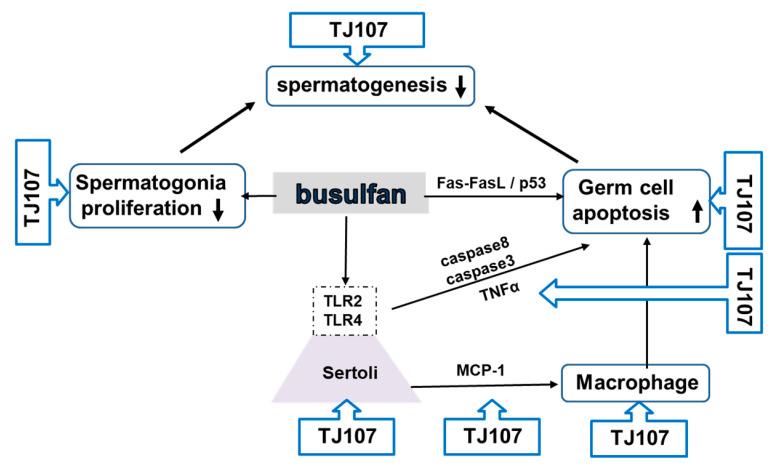
Immune mechanisms of Goshajinkigan in infertility after BSF treatment. ↑ indicates an increase and ↓ indicates a decrease. Abbreviations: macrophage chemotactic protein: MCP; Toll-like receptors: TLR.

In pediatric patients treated with busulfan (BSF; Myleran, 1,4-butanediol dimethanesulfonate), an alkylating agent used for bone marrow transplantation [87], the recovery of spermatogenesis is a slow and progressive process [88,89,90,91]. Given that BSF induces the death and depletion of spermatogonia [92,93,94], it has been widely utilized to prepare testicular recipients for spermatogonia stem cells transplantation [90,95,96]. The expression of proliferation-related genes was significantly decreased by BSF treatment, while apoptotic genes (*Fas*, *FasL*, *Caspase3*, and *Caspase8*) were significantly increased. Furthermore, BSF-induced spermatogenic cell damage upregulated Toll-like receptor (TLR) 2 and TLR4 expressions in Sertoli cells and facilitated macrophage infiltration into the testis (see Table 1 and Figure 3). Despite considerable efforts to improve spermatogenesis following BSF disruption, there is currently no effective treatment for this condition. In Japan, traditional herbal medicines have been approved for clinical use by the National Health Insurance Program and are used to treat male infertility. One such herbal medicine, Goshajinkigan (TJ107), composed of 10 herbal ingredients (see Table 2), has been widely used in Japan and China to treat conditions such as meralgia, lower back pain, numbness, and neuropathy, particularly in elderly patients. Recently, we demonstrated that TJ107 was able to completely recover the injured seminiferous epithelium by normalizing macrophage migration and reducing the expression of TLR 2 and 4, and promote recovery from severe aspermatogenesis following BSF treatment in mice [26,27]. This suggests that TJ107 has a therapeutic effect on BSF-induced infertility (see Table 1 and Figure 3).

**Figure 4:**
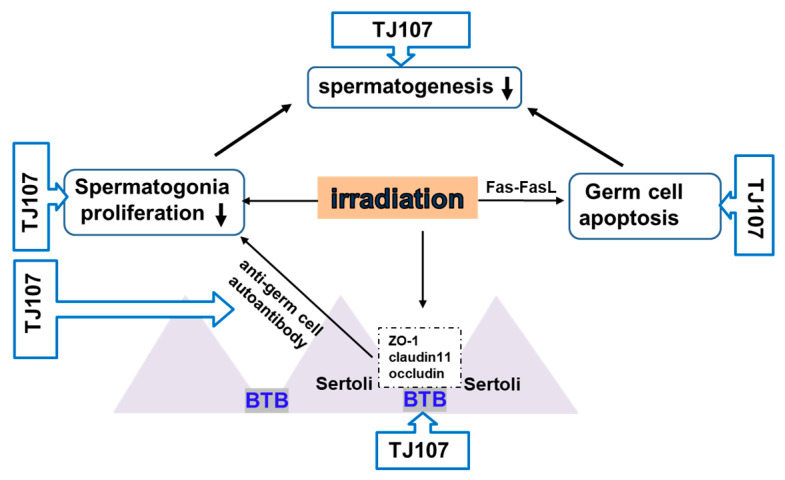
Immune mechanisms of Goshajinkigan in infertility after irradiation treatment. ↑ indicates an increase and ↓ indicates a decrease. Abbreviations: blood–testis barrier: BTB.

Importantly, our examination further demonstrated that impaired reproductive function induced by cancer treatment, including chemotherapy and radiotherapy, is associated with various pathophysiological conditions [17]. In particular, examination detected that ASA production and inter-Sertoli tight-junction barrier disruption were induced by the 6Gy of total body irradiation. The breakdown of the BTB tight junction due to irradiation may cause repeated leakage of germ cell autoantigens within the BTB, leading to a continuous supply of autoantigens for immune stimulation. This process results in the production of ASA and prolongs testicular inflammation [17]. Supplementation with TJ107 restored disrupted inter-Sertoli tight junctions by normalizing the expression of claudin-11, occludin, and ZO-1, while also reducing serum ASA levels [29] (see Table 1 and Figure 4). Our studies demonstrated that impaired reproductive function induced by cancer treatments, including chemotherapy and radiotherapy related to different immune-pathophysiological conditions, can be cured by TJ107. We will examine the effects of other traditional Japanese medicines, such as Hachimijiogan and Hochuekkito, on oncologic aspermatogenesis and evaluate the efficacy of a polyherbal formulation for improving fertility after cancer treatment in the next experiments.

Currently, there is limited information regarding therapies for anticancer-induced male infertility in Western medicine. These four oriental medicines (TJ107, MYOMI-7, QJT, and SJD) examined proved the effective treatment in anticancer-induced aspermatogenesis in an animal model, although the impaired reproductive function is associated with various pathophysiological conditions. These oriental medicines promote recovery from severe aspermatogenesis by reducing germ cell apoptosis and/or normalizing BTB dysfunction. It is worth noting that *Rehmanniae radix (RR)*, widely used in clinical settings, is included in the three oriental medicines as TJ107, QJT, and SJD (see Table 2). RR’s pharmacological actions span multiple systems, offering antioxidant, immune-modulating, anti-inflammatory, and anti-aging benefits, and are principally applied in the treatment of gynecological and diabetic metabolic disorders, cardiovascular diseases, and osteoporosis [15]. RR could possess the ability to alleviate heat, promote blood cooling, enhance kidney Yin and fluid nourishment, and has become a common treatment in clinical settings due to its protective effects on the testis. On the other hand, *Plantaginis semen (PS)* has diuretic, anti-inflammatory, hypoglycemic, hypolipidemic, and antioxidant effects, and enhances kidney Yang. In TJ107 and QJT, RR and PS are used together, which shows superior results in recovering insufficient kidney Yang, impairing the kidney Yin’s ability.

## 4. Limitations of Studies on Oriental Medicines

Oriental medicine has a long history. The history of medical development shows that oriental medicine, or traditional medicine, was born through medical practice during the times when science and technology were immature and underdeveloped. So, oriental medicine is primarily applied as a clinical treatment, and its chemical and pharmacological foundations are not well understood in most cases. Since oriental medicine is an empirical treatment based on the “practitioner’s experience”, and there are also reports that it is ineffective, the pharmacological effect remains unclear. In cryptozoospermic patients with varicocele reports, Liu et al. [49] applied JWRJD to 58 patients and demonstrated that JWRJD promoted spermatogenesis, especially for patients > 35 years old (n = 14) and having grade III varicocele (n = 13). The large numbers of patients in the group ≤35 years old (n = 44) and having grade 0~II varicocele (n = 45) showed different effects after JWRJD treatment. Although they demonstrated that JWRJD could significantly reduce FSH levels in older men and promote spermatogenesis, the fundamental difference in pharmacological effect has yet to be clarified. On the other hand, there are some case reports based on kidney Yang vacuity (fatigue, chills, pale, and faint pulse) and kidney Yin deficiency (light-headed, tinnitus, dark red tongue, and strong pulse), but not by pathological examination and western medical diagnosis. Chen and Wen [97] conducted a nonrandomized study on male infertility in 202 patients using *Sheng Jing*, a Chinese herbal formula. Seventy-seven percent of the patients were diagnosed under the pattern of kidney Yang vacuity, and thirty-three percent of the patients were characterized as kidney Yin deficiency. They reported significant improvements in sperm density, motility, and grade; the levels of FSH, LH, and testosterone; and a reduction in serum ASA titers.

Furthermore, there are many case reports about oriental medicine written in the local language, but there are still very few original research papers written in English, and even fewer on male infertility, which is a rare area of research. More experimental and clinical studies using modern scientific principles and methods in this field are recommended. The above research mentioned in this review provides some preliminary insights into the pharmacological basis and molecular immune mechanisms of oriental medicines for treating male infertility; further experimental analysis and research are required to validate these findings. According to the pharmacological effect on male infertility and testicular immune mechanisms, some oriental medicines are expanding the scope of treatment, such as the implications of chromosomal abnormalities, such as Klinefelter syndrome (47,XXY), which not only represent a common genetic cause of male infertility but are also associated with an increased risk of autoimmune diseases [98]. Combined application with Western medicines and more publications are needed to improve oriental medicines developed into clinical drugs, evaluate the safety, and achieve international recognition/availability.

## 5. Conclusions

Oriental medicine has gained attention and is increasingly being utilized as a complementary therapy in infertility. There is limited information regarding the testicular immune response, and understanding the mechanisms of male infertility is essential for advancing treatment. From the above limited reported data, further experimental research into oriental medicines may verify various abnormalities in immune-related cells or active molecules associated with infertility and the molecular immune mechanisms underlying these findings. This review summarized some infertility types with insufficient treatment effects in Western medicine and examined studies evaluating the use of traditional herbal formulas rather than individual herbal medicines in treating the repair of Kidney Yin–Yang. The other mechanisms that could be activated by the substances present in the individual herbal plants used in infertility treatments will be discussed in a future study. Empirical oriental medicine treatment, which is prescribed based on the experience of clinicians, is expected to create an academic foundation for oriental medicine treatment for male infertility, and collaboration between modern medicine and traditional medicine should be fostered to promote the advancement of knowledge and patient care.

## Figures and Tables

**Table 1:** Immune factors in the normal and infertile testes.

	Immuno- Regulatory Factors in Normal Testis	Anti-Cancer Treatment	Varicocele	Oxidant Stress	IOA
germ	TGF-β		↑ [22]	↑ [23]	↑ [24]
cells	Fas-L	↑ [25,26,27]	↑or (-) [22]		↑ [24]
	IFN-γ		↑ [22]		
	TNF-α	↑ [25,26,27]	↑ [22]	↑ [23,28]	
		Fas↑ Bax↑ [25]	Fas↑ [22]	caspase3↑ [28]	PI3K/AKT/mTOR↓ [24]
		Caspase3,8↑ [25,26,29]	Caspase1↑ [30]	Bax↑ p53↑ [31,32,33]	ROS↑ [34]
		p53-ROS↑ [25]		SOD↑ [31]	
Sertoli	activin				
cells	inhibin				
	IL-6		↑ [22]	↑ [35]	
	Fas-L	↑ [26,27]			↑ [24]
	TNF-α	↑ [25,26,27]	↑ [22]	↑ [35]	
		MCP-1↑ [26,27]	claudin-11↓ [22]		
		TLR2,4↑ [26,27]			
		Occludin↓ [29,36]			
		ZO-1↓ [29,36]			
		F-actin↓ [36]			
Leydig	testosterone	↓ [36]	↓ [22,37]	↓ [33]	↓ [38]
cells	protein s				
	Fas-L				
	IL-10		↑ [22]		
	TGF-β		↑ [22]		
		Bcl-2↑ [25]			
Testicular	IL-10		↑ [22]		↑ [24]
macro-	IFN-γ		↑ [22]		
phages	IL-6		↑ [22]	↑ [31,32]	
	TNF-α	↑ [26,27]	↑ [22]	↑ [35]	
		macrophage infiltration↑ [26,27]	IL-1β ↑ [22,39]	IL-1β↑ [35]	
Others		FSH, LH↓ [40]	ASA↑ [22]	ROS/NOS↑ [23,28,31,32,33,41,42]	MMP↓ [43,44,45,46]
		MMP↓ [47]	ROS↑ [30,48]	FSH, LH↓ [42]	
		ROS, MDA↑ [25,40]	FSH ↑ [49]		
		ASA↑ [21,29]	NLRP3↑ [22,39]		

↑ indicates an increase and ↓ indicates a decrease. Abbreviations: anti-sperm antibodies: ASA; B-cell lymphoma 2: Bcl-2; Bcl-2 associated X protein: Bax; Fas ligand: Fas-L; follicle-stimulating hormone: FSH; interferon-γ: IFN-γ; interleukin: IL; luteinizing hormone: LH; macrophage chemotactic protein: MCP; malondialdehyde: MDA; mitochondrial membrane potential: MMP; nucleotide-binding oligomerization domain-like receptor family pyrin: NLRP; nitric oxide synthase: NOS; reactive oxygen species: ROS; superoxide dismutase: SOD; transforming growth factor β: TGF-β; tool-like receptor: TLR; tumor necrosis factor α: TNF-α.

## Data Availability

Not applicable.

## Abbreviations

Acute myelogenous leukemia: AML; anti-sperm antibodies: ASA; B-cell lymphoma 2: Bcl-2; Bcl-2 associated X protein: Bax; busulfan: BSF; blood–testis barrier: BTB; cyclophosphamide: CP; estrogen receptor: ER; Fibrous actin: F-actin; Fas ligand: Fas-L; follicle-stimulating hormone: FSH; gonadotropin-releasing hormone: GnRH; intracytoplasmic sperm injection: ICSI; interferon-γ: IFN-γ; interleukin: IL; luteinizing hormone: LH; idiopathic oligoasthenozoospermia: IOA; macrophage chemotactic protein: MCP; malondialdehyde: MDA; mitochondrial membrane potential: MMP; nucleotide-binding oligomerization domain-like receptor family pyrin: NLRP; nitric oxide synthase: NOS; reactive nitrogen species: RNS; reactive oxygen species: ROS; Selective estrogen receptor modulators: SERMs; superoxide dismutase: SOD; transforming growth factor β: TGF-β; tool-like receptor: TLR; tumor necrosis factor α: TNF-α.
